# High humidity environment increases FBG by impairing the intestinal barrier

**DOI:** 10.3389/fimmu.2025.1625609

**Published:** 2025-08-27

**Authors:** Yao Wang, Kai Zhuang, Qiuxia Yi, Yalan Wu, Yi Luo, Yulin Ouyang, Liang Li, Chun Li, Huanhuan Luo

**Affiliations:** ^1^ State Key Laboratory of Traditional Chinese Medicine Syndrome, Guangzhou University of Chinese Medicine, Guangzhou, Guangdong, China; ^2^ Chinese Medicine Guangdong Laboratory, Hengqin, Guangdong, China; ^3^ Research Centre of Basic Integrative Medicine, School of Basic Medical Sciences, Guangzhou University of Chinese Medicine, Guangzhou, Guangdong, China; ^4^ Zhongshan Hospital of Traditional Chinese Medicine Affiliated to Guangzhou University of Traditional Chinese Medicine, Zhongshan, Guangdong, China; ^5^ The Affiliated Hospital of Liaoning University of Traditional Chinese Medicine, Shenyang, Liaoning, China; ^6^ School of Pharmaceutics, Guangzhou University of Chinese Medicine, Guangzhou, Guangdong, China

**Keywords:** high humidity environment, intestinal barrier, gamma-aminobutyric acid, glucagon, fasting blood glucose

## Abstract

**Introduction:**

Climate and environmental changes pose significant threats to human metabolic health; however, the specific effects of individual environmental factors on metabolic diseases remain poorly understood. This study aimed to investigate the impact of a high humidity environment (HH) on fasting blood glucose (FBG), intestinal barrier integrity, and gut microbiota composition.

**Methods:**

We analyzed clinical samples collected during HH exposure and performed a controlled male mouse experiment. FBG and hormone levels were assessed, and intestinal barrier integrity was evaluated using western blot and immunofluorescence staining. Gut microbiota composition was profiled via 16S rDNA sequencing. Mechanistic insights were obtained through fecal microbiota transplantation (FMT), Mendelian randomization (MR) analysis, and metabolomic profiling. An antibiotic cocktail (ABX) intervention was applied to determine the reversibility of HH-induced effects.

**Results:**

Clinical samples collected under HH conditions showed elevated FBG, increased glucagon (GC) levels, impaired intestinal barrier function, and decreased serum gamma-aminobutyric acid (GABA) concentrations. 16S rDNA sequencing revealed increased abundances of *Alistipes, Parabacteroides*, and *Akkermansia*. Metabolomic analysis demonstrated reduced serum GABA levels, which correlated with intestinal barrier disruption and activation of the MDP-NOD2 pathway in pancreatic β-cells. HH exposure also downregulated GAD67 expression, reducing GABA synthesis and leading to enhanced GC secretion from islet α-cells. FMT suggested that gut microbiota alterations mediated HH-induced FBG elevation. ABX treatment effectively reversed these metabolic and microbial changes.

**Discussion:**

Our findings demonstrate that a high humidity environment disrupts gut microbiota homeostasis, impairs the intestinal barrier, and reduces GABA synthesis in pancreatic β-cells, thereby promoting the development of type 2 diabetes mellitus (T2DM).

## Graphical Abstract

High humidity promotes T2DM progression by impairing intestinal barrier integrity, altering gut microbiota composition, and disrupting GABA-mediated regulation of glucagon secretion.

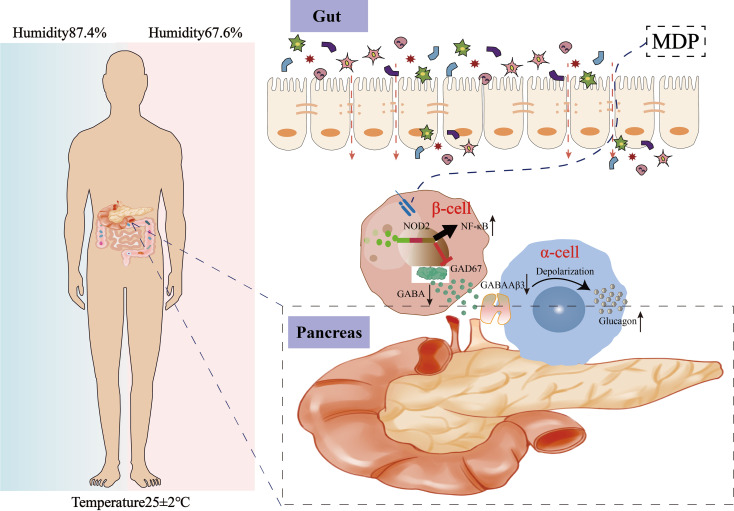

## Introduction

The Intergovernmental Panel on Climate Change forecasts that episodes of humidity and heat stress will become more intense and frequent in the years ahead (1). A regional study also reported a higher prevalence of diabetes among elderly individuals residing in areas with elevated relative humidity (2). However, the mechanisms by which climatic changes, such as shifts in humidity, contribute to metabolic disorders are still not well understood.Type 2 diabetes mellitus (T2DM) is a progressive metabolic disorder characterized by dysfunction of pancreatic β-cells and peripheral insulin resistance, leading to impaired glucose metabolism and chronic low-grade inflammation. The onset of T2DM is intricately linked to both genetic and environmental factors, with environmental elements such as calorie intake, nutrient composition, ambient air pollution, and physical inactivity significantly contributing to the rising incidence of the disease (3). The mechanisms by which high humidity environment contribute to metabolic disorders remain poorly understood, and effective intervention strategies are still needed. Intestinal barrier dysfunction, leading to the migration of bacteria and their products, is increasingly recognized as a key factor in the development of T2DM (4–6). A compromised barrier can increase intestinal permeability, allowing microbiota-derived endotoxins such as lipopolysaccharide (LPS) and muramyl dipeptide (MDP) to enter the bloodstream (7). The intestinal barrier plays a crucial role in diseases driven by environmental changes, either mitigating or worsening the effects on host health. Variations in environmental humidity may alter the gut microbiota in mammal, with microbial adjustments affecting various physiological and pathological processes. The presence of MDP, a significant element of bacterial cell walls in the gut, activates the nucleotide-binding oligomerization domain 2 (NOD2) (8), which triggers a proinflammatory signaling cascade that contributes to insulin resistance (9). Therefore, therapies or agents that enhance intestinal barrier function could offer new avenues for preventing and treating T2DM. Gamma-aminobutyric acid (GABA) is a major inhibitory neurotransmitter in the central nervous system (CNS) and is distributed widely in various host tissues and their receptors. Besides the CNS, GABA is also found in numerous tissues (10). Remarkably, the highest concentration of GABA, outside the brain tissue, is located in pancreatic islets (11). In T2DM, GABA has been shown to preserve β-cell mass and prevent diabetes (12). Commensal microbes significantly influence host metabolism (13), making it crucial to understand their regulatory roles. These microbes aid in the digestion of otherwise indigestible dietary components, synthesize essential metabolites, and modulate host metabolism by interacting with the intestine and distant organs. Previous studies have shown the importance of the intestine-islet axis for host glucose tolerance, where beta cells detect microbial Nod1 ligands released by commensal bacteria through intestinal lysozyme activity (14). Based on this, it is hypothesized that gut microbes may cross the intestinal barrier, influencing pancreatic beta-cell synthesis of GABA.In this study, we explored the effect of HH environment on T2DM and intestinal barrier integrity. Our findings suggest that the HH environment impares the intestinal barrier and GABA production, leading to an increase in GC and FBG, thereby accelerating the progression of T2DM, while antibiotic treatment mitigates these effects. These results highlight the influence of HH conditions on T2DM and its mechanisms, aiding in the development of targeted intervention strategies.

## Results

### High humidity environment increases FBG and GC in T2DM patients, related to disruption of gut microbiota and intestinal barrier

**Figure 1:**
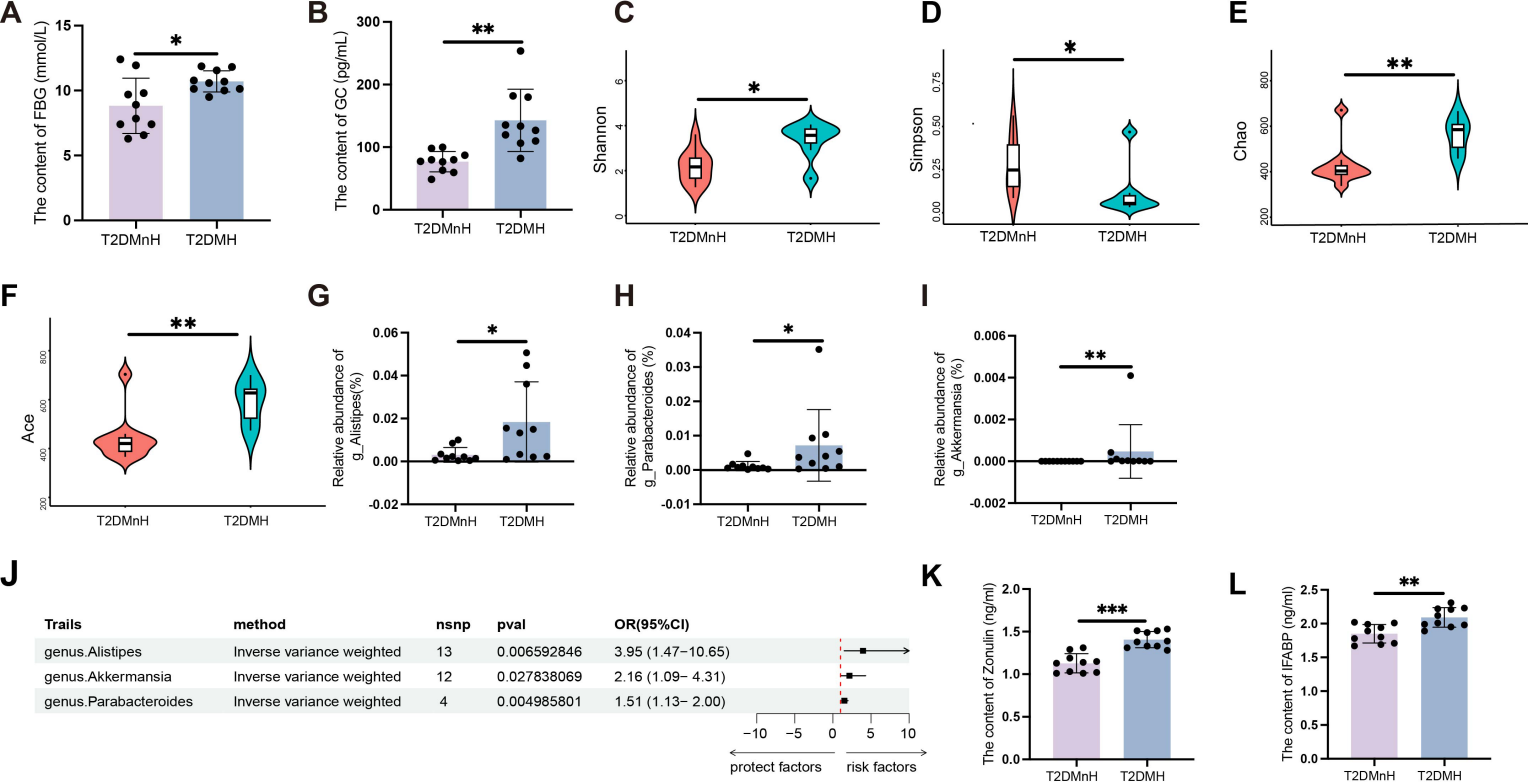
High humidity environment increases FBG and GC in T2DM patients, related to disruption of gut microbiota and intestinal barrier. **(A, B)** The content of FBG and GC between the T2DMnH and T2DMH groups. n = 10, **(A, B)** were analyzed by student’s two-tailed t test. **(A)** t=2.609, *P* =0.018. **(B)** t=3.988, *P* =0.0009. **P < 0.01, *P < 0.05. **(C-I)** The Shannon **(C)**, Simpson **(D)**, Chao **(E)** and Ace **(F)** indices between the T2DMnH and T2DMH groups. The relative abundance of genus *Alistipes* **(G)**, *Parabacteroides* **(H)**, and *Akkermansia* **(I)**. n = 10, **(C-I)** were analyzed by Mann-Whitney U. **(C)** U=19, *P* =0.019. D: U=21, *P* =0.029. **(E)** U=14, *P* =0.005. **(F)** U=14, *P* =0.005, **(G)** U=18, *P* =0.015, **(H)** U=21, *P* =0.029, **(I)** U=22, *P* =0.004. **P < 0.01, *P < 0.05. **(J)** The forest plot shows the causal estimates between microbial taxa at the genus level and T2DM. The OR and 95% CI were obtained using the inverse variance weighted method. **(K-L)**: The content of zonulin and IFABP. n = 10, **(K, L)** were analyzed by student’s two-tailed t test. **(K)** t=5.979, **(L)** t=3.826, *P* =0.0012. ***P < 0.001, **P < 0.01. Data are shown as means ± SD. FBG, Fasting Blood Glucose; GC, Glucagon; IFABP, Intestinal Fatty Acid Binding Protein.

Previous studies by our research team have shown that humid and high-temperature environments can lead to gut microbiota dysbiosis, which in turn affects GLP-1 (15). To examine the metabolic effects of HH environment, serum and fecal samples were collected from male T2DM patients. Serum samples were analyzed for glucose and lipid profiles. The results indicated that FBG levels in the T2DMH group were significantly higher than those in the T2DMnH group (**Figure 1A**, P < 0.05), while no statistically significant differences were observed in postprandial blood glucose (PBG) levels (**Supplementary Figure S1A**).

Additionally, levels of triglycerides (TG) (**Supplementary Figure S1B**), cholesterol (CHOL) (**Supplementary Figure S1C**), high-density lipoprotein (HDL) (**Supplementary Figure S1D**), and low-density lipoprotein (LDL) (**Supplementary Figure S1E**) did not show significant differences. Glucagon (GC) and insulin (INS), key hormones in glucose regulation, were also measured. A significant increase in GC levels was observed in the T2DMH group (**Figure 1B**, P < 0.01), whereas INS levels remained unchanged (**Supplementary Figure S1F**). Moreover, the content of glucagon-like peptide-1 (GLP-1) (**Supplementary Figure S1G**), peptide YY (PYY) (**Supplementary Figure S1H**), gastric inhibitory polypeptide (GIP) (**Supplementary Figure S1I**) and Ghrelin (**Supplementary Figure S1J**) also showed no obvious changed. In contrast to humid and high-temperature environments, glucagon GC exhibited a significant change in HH environments, while other hormones, such as GLP-1, showed no variations. This indicates that the upregulation of GC in HH environments is a major factor contributing to the increase in blood glucose levels.

To assess bacterial diversity between the groups, sequence data were aligned to estimate alpha diversity indices. Statistically significant differences were found in the Shannon (**Figure 1C**, P < 0.05), Simpson (**Figure 1D**, P < 0.05), Chao (**Figure 1E**, P < 0.01), and Ace (**Figure 1F**, P < 0.01) indices, indicating higher alpha diversity in the T2DMH group compared to T2DMnH. Additionally, the abundance of the genus *Alistipes* (**Figure 1G**, P < 0.05), *Parabacteroides* (**Figure 1H**, P < 0.05), and *Akkermansia* (**Figure 1I**, P < 0.01) were elevated in the T2DMH group. Utilizing Mendelian randomization (MR) analysis, to infer potential causal relationships, we identified increased gut microbiota genus *Alistipes, Parabacteroides*, and *Akkermansia* as risk factors for T2DM (**Figure 1J** and **Supplementary Table S1**, *P* < 0.05).

Disruption of the gut microbiota can lead to damage to the intestinal barrier (16). In T2DM patients under HH environment (T2DMH), serum zonulin levels were significantly higher (**Figure 1K**, P < 0.001). Intestinal fatty acid-binding protein (IFABP), which regulates fatty acid metabolism and is exclusively expressed in intestinal epithelial cells, typically remains at low concentrations under normal conditions. However, IFABP is released into the circulation following damage to intestinal epithelial cells (17). Elevated serum IFABP levels were also observed in T2DMH patients (**Figure 1L**, P < 0.01). These findings suggest that a HH environment may disrupt the gut microbiota of T2DM patients, impair the intestinal barrier, and lead to increased GC and FBG levels.

### Transplantation of the faecal microbiota from the HH group recapitulates alterations in the intestinal barrier and increase of FBG in DF mice

In order to further verify the mechanisms by which a humid environment induces elevated blood glucose levels, we carried out experiments on animals. Adult male BALB/c mice were housed either in an ordinary environment (the NC group, 25 ± 1°C and 60–65% humidity) or a HH environment (the HH group, 25 ± 1°C and 90–95% humidity) for 28 days, after which they underwent a series of glucose and lipid tests.

**Figure 2:**
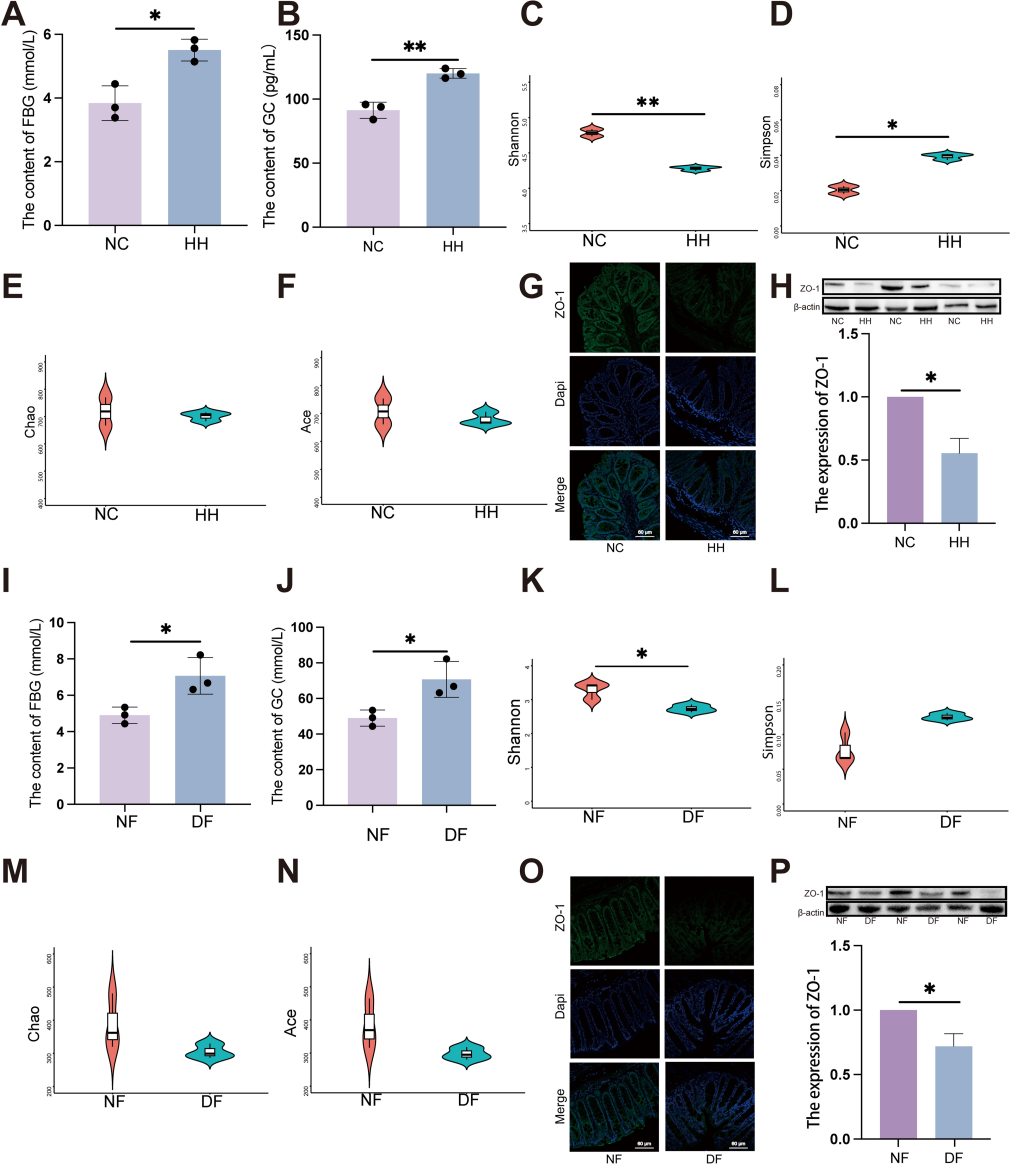
Transplantation of the faecal microbiota from the HH group recapitulates alterations in the intestinal barrier and increase of FBG in DF mice. **(A)** FBG level in NC and HH group (n = 3). **(A)** was analyzed by student’s two-tailed t test, t=4.490, *P* =0.011. *P < 0.05. **(B)** GC level in NC and HH group (n = 3). **(B)** was analyzed by student’s two-tailed t test, t=6.804, *P* =0.002. **P < 0.01. **(C-F)** Species diversity differences between the NC and HH groups were estimated by the Shannon **(C)**, Simpson **(D)**, Chao **(E)** and Ace **(F)** indices (n = 3). **(C)** was analyzed by student’s two-tailed t test, t=5.806, *P* =0.004. **(D)** was analyzed by student’s two-tailed t test, t=3.824, *P* =0.019. **P < 0.01, *P < 0.05. **(G, O)** IF of ZO-1. The green represents ZO-1 and the blue represents Dapi. Scale bars=60μm. **(H, P)** The expression of ZO-1 by western blot. **(H, P)** were analyzed by Welch’s t test. **(H)** t=6.530, *P* =0.023. **(P)** t=4.994, *P* =0.038. *P < 0.05. **(I)** FBG level in NF and DF group (n = 3). **(I)** was analyzed by student’s two-tailed t test, t=3.406, *P* =0.027. *P < 0.05. **(J)** GC level in NF and DF group (n = 3). **(J)** was analyzed by student’s two-tailed t test, t=3.406, *P* =0.027. *P < 0.05. **(K-N)** Species diversity differences between the NF and DF groups were estimated by the Shannon **(K)**, Simpson **(L)**, Chao **(M)** and Ace **(N)** indices (n = 3). **(I)** was analyzed by student’s two-tailed t test, t=3.50, *P* =0.029. *P < 0.05. Data are shown as means ± SD. FBG, Fasting Blood Glucose; GC, Glucagon.

Consistent with clinical samples, significant elevation in FBG (**Figure 2A**, P < 0.05) and GC (**Figure 2B**, P < 0.01) levels were observed under HH environment. The levels of PBG (**Supplementary Figure S2A**), TG (**Supplementary Figure S2B**), CHOL (**Supplementary Figure S2C**), HDL (**Supplementary Figure S2D**) and LDL (**Supplementary Figure S2E**) in the HH group did not show significant changes compared to the NC group.

Given that the HH environment impacts blood glucose levels rather than blood lipids, we therefore assessed the gastrointestinal hormones that regulate blood glucose fluctuations. Nevertheless, within the BALB/c mouse model, it was observed that the content of INS (**Supplementary Figure S2F**), GLP-1 (**Supplementary Figure S2G**), PYY (**Supplementary Figure S2H**), GIP (**Supplementary Figure S2I**) and Ghrelin (**Supplementary Figure S2J**) remained no obvious change between NC and HH group.

Significant alterations in the composition of the gut microbiota were detected through 16S rDNA gene sequencing of fecal samples, particularly notable were the marked differences in α diversity, including observed Shannon and Simpson indices (**Figures 2C, D**, P < 0.05). However, Chao and Ace indices showed no significant changes (**Figures 2E, F**). Furthermore, both immunofluorescence and western blot (WB) analyses revealed a significant reduction in ZO-1 expression levels in the HH group compared to the NC group (**Figures 2G, H**). This suggests that in HH environment, disruption of the gut microbiota is accompanied by impaired intestinal mucosal barrier function.

Germ-free mice received FMT *via* oral gavage using mouse stools from either the HH group or the NC group, designated as the DF and NF groups respectively, to confirm the direct impact of gut microbiota induced by HH environment (**Supplementary Figure S2K**). Fecal samples were collected from the DF and NF groups 28 days post-gavage, followed by 16S rDNA sequencing. Consistent with the results from the HH group, the FBG levels were also significantly higher in the DF group compared to the NF group (**Figure 2I**, P < 0.05). GC levels were significantly elevated in the DF group (**Figure 2J**, P < 0.05). The findings from fecal microbiota transplantation further corroborate that HH environment exerts an influence on the homeostasis of the associated gut microbiota. Comparisons of bacterial abundance between the DF group and the NF group, as well as the HH group and the NC group, revealed similar changes, indicating successful colonization. Compared to the NF group, the alpha diversity of the gut microbiota in the DF group was significantly decreased, mirroring that observed in the HH group. Specifically, the Shannon index showed a significant decrease (**Figure 2K**, P < 0.05), while the Simpson, Chao, and Ace indices remained relatively unchanged (**Figures 2L-N**). In DF groups, ZO-1 expression was significantly reduced compared to NF groups, as indicated by immunofluorescence staining (**Figure 2O**, P < 0.05) and WB analysis (**Figure 2P**). Collectively, these findings suggest that a HH environment compromises the intestinal barrier, elevates FBG levels, and that alterations in the gut microbiota play a crucial role in these processes.

### Gut microbiota dysbiosis induced by high humidity environment contributes to impairs intestinal barrier and T2DM development

**Figure 3:**
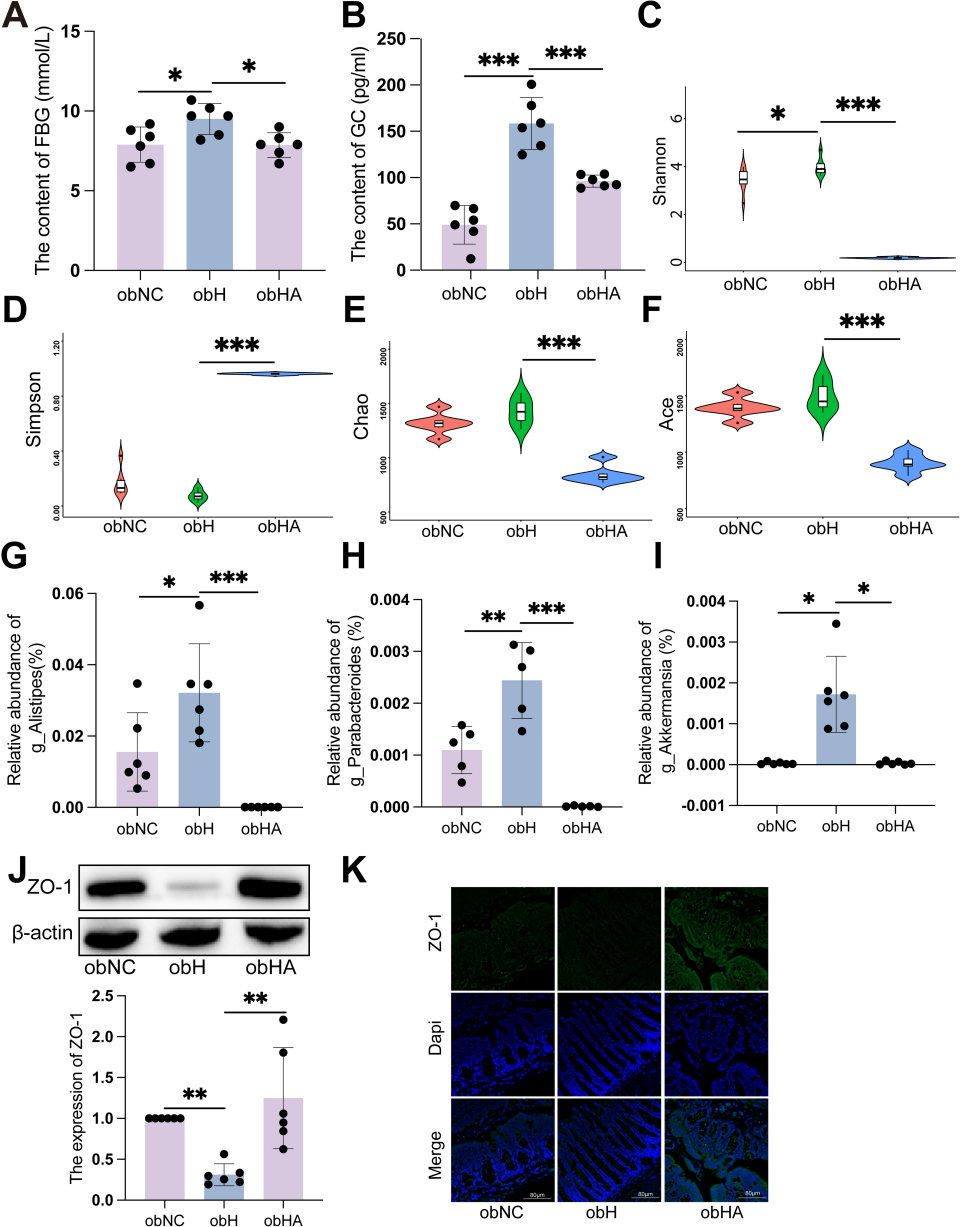
Gut microbiota dysbiosis induced by high humidity environment contributes to impairs intestinal barrier and T2DM development. **(A)** The level of FBG in different groups (n = 6). **(A)** was analyzed by Tukey test, F=0.620, P =0.015. *P < 0.05. **(B)** The level of GC in different groups (n = 6). **(B)** was analyzed by Tukey test, F=2.280, ***P < 0.001. **(C-F)** Alpha diversity represented by Shannon **(C)**, Simpson **(D)**, Chao **(E)** and Ace **(F)**. n = 6, **(C-F)** were analyzed by Tukey. **(C)** F=3.513, **(D)** F=2.228, **(E)** F=1.090, **(F)** F=1.151, ***P < 0.001, *P < 0.05. **(G-I)** The relative abundance of genus Alistipes **(G)**, Parabacteroides **(H)**, and Akkermansia **(I)**. n = 6, **(G, H)** were analyzed by Tukey. **(G)** F=3.085, **(H)** F=3.670, **(I)** were analyzed by Kruskal-Wallis test. **(I)** K=19.21, ***P < 0.001, **P < 0.01, *P < 0.05. **(J)** The expression of ZO-1 by western blot. n = 6, K was analyzed by Kruskal-Wallis test. **(J)** K=11.79, **P < 0.01. **(K)** IF of ZO-1. The green represents ZO-1 and the blue represents Dapi. Scale bars=60μm. Data are shown as means ± SD. FBG, Fasting Blood Glucose; GC, Glucagon.

Exposure to HH environment results in elevated blood glucose levels in BALB/c mice. However, to elucidate more clearly the relationship between HH environment and diabetes, we subsequently conducted a series of assessments using the diabetic *ob/ob* mouse model. The level of FBG in *ob/ob* mice under HH environment (obH) was higher than that in normal environment (obNC) (**Figure 3A**, P < 0.05). Moreover, bacteria in the intestinal tract were depleted by the administration of an antibiotic cocktail (penicillin, 1 mg/mL; neomycin, 1 mg/mL; metronidazole, 0.5 mg/mL; vancomycin, 0.5 mg/mL). In *ob/ob* mice treated with antibiotics (obHA), FBG levels were significantly lower compared to those in untreated *ob/ob* mice under HH environment (obH) (**Figure 3A**, P < 0.05). However, changes in PBG were not statistically significant (**Supplementary Figure S3A**). Consistent with clinical findings, we observed a significant increase in GC levels in the obH group, which decreased after antibiotic treatment (**Figure 3B**, P < 0.001), while INS levels showed no significant changes (**Supplementary Figure S3B**). To investigate the potential role of gut microbial dysbiosis in the development of T2DM associated with HH environment, we conducted 16S rDNA sequencing on stool samples from obNC, obH, and obHA mice. Differences in microbiota composition were evident among these groups using alpha diversity analysis. The Shannon index was significantly higher in obH mice compared to obNC (**Figure 3C**, P < 0.05), indicating greater bacterial diversity in the obH group. No significant differences were found in the Simpson, Chao, and Ace indices (**Figures 3D-F**). Consistent with these findings, several bacterial taxa were differentially abundant in mice exposed to the HH environment. The abundances of potential pathogenic bacterial species including genus *Alistipes* (**Figure 3G**, P < 0.05), *Parabacteroides* (**Figure 3H**, P < 0.01), and *Akkermansia* (**Figure 3I**, P < 0.05) were significantly higher in obH mice than obNC mice.

To test whether gut barrier function plays a role in HH-associated T2DM, we examined the impact of HH on paracellular permeability in the colon of mice by measuring the expression of ZO-1. WB and IF showed that the expressions of tight junctional proteins ZO-1 were significant reduced in obH mice, and were significant increased in obHA mice (**Figures 3J, K**, P < 0.05). Taken together, these results indicated that HH causes impaired colonic barrier function and development of T2DM at least in part through causing gut microbial dysbiosis.

## High humidity environment inhibited the expression of GAD67 in pancreatic β-cell by modulating gut microbiota

**Figure 4:**
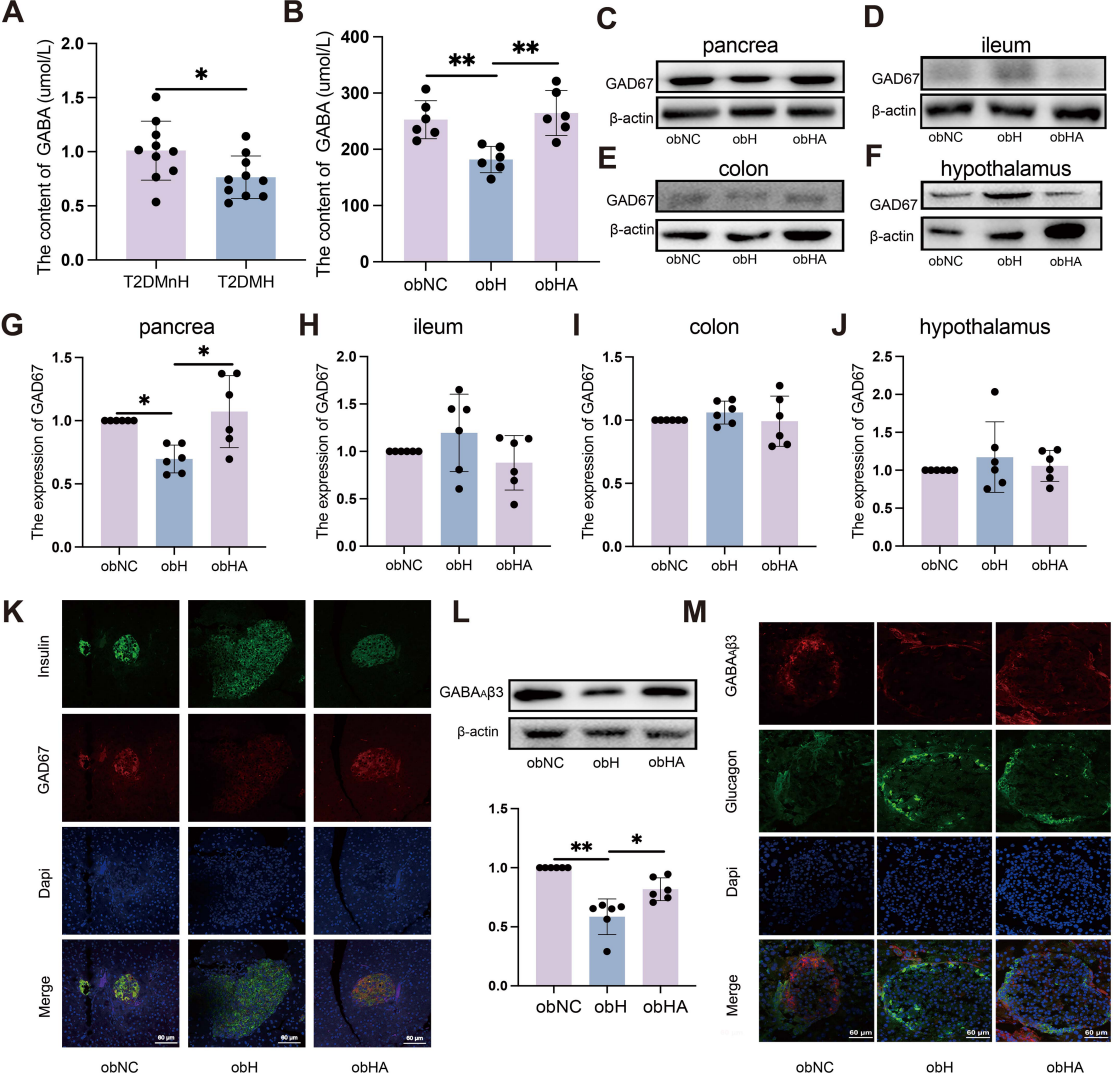
High humidity environment inhibited the expression of GAD67 in pancreatic β-cell by modulating gut microbiota. **(A)** Results from MRM analysis of neurotransmitter levels in the blood of clinical patients (n = 10). **(A)** t=2.318, *P* = 0.0324. *P < 0.05. **(B)** The content of GABA (n = 6). **(B)** was analyzed by Tukey, F=10.97, *P* = 0.0012. **P < 0.01. **(C–J)** Western blots of GAD67 in pancrea **(C, G)**, ileum **(D, H)**, colon **(E, I)** and hypothalamus **(F, J)**. **(C)** was analyzed by Kruskal-Wallis test. **(C)** K=9.938, *P* = 0.0023. *P < 0.05. **(K)** IF of GAD67 in pancreatic β-cells. The green represents pancreatic β-cells, the red represents GAD67 and the blue represents Dapi. Scale bars=60μm. **(L)** Western blot analysis of GABA_A_β3 expression. n = 6, obNC VS obH was analyzed by Welch’s t test, t=6.748, *P* =0.001. obH VS obHA was analyzed by Welch’s t test, t=3.185, *P* =0.012. **P < 0.01, *P < 0.05. **(M)** IF staining of GABA_A_β3 in pancreatic α-cells, with green indicating pancreatic α-cells, red for GABA_A_β3, and blue for Dapi nuclear staining. Scale bars=60μm. Data are shown as means ± SD. GABA, Gamma-Aminobutyric Acid; GAD67, Glutamate Decarboxylase 67; GABA_A_β3, Gamma-Aminobutyric Acid Receptor Subunit Beta-3.

The HH environment impacts GC and FBG levels, yet does not appear to affect insulin secretion. Consequently, we hypothesize that the gut microbiota may exert an influence on the functionality of pancreatic alpha and beta cells through the impairment of intestinal barrier integrity. Emerging research highlights the role of neurotransmitters in diabetes treatment, including GABA (18). Additionally, studies have substantiated that the synthesis of neurotransmitters such as GABA may be modulated by gut microbiota (19). GABA possesses the capacity to concurrently modulate the functions of pancreatic α and β cells, and it similarly exhibits a regulatory influence on GC (20, 21). Consequently, to elucidate the specific mechanisms through which the HH environment influences GC and FBG, the Multiple Reaction Monitoring (MRM) method was employed to measure neurotransmitter levels in the serum of T2DM patients. Among tested neurotransmitters, GABA showed statistically significant variations between T2DMH and T2DMnH groups, specifically displaying a significant reduction in the T2DMH group (**Figure 4A**, **Supplementary Figure 4**, *P* < 0.05). A similar trend was observed in *ob/ob* mice, where GABA levels were significantly decreased in the obH group and increased following antibiotic treatment (**Figure 4B**, P < 0.05).

Gutamate decarboxylase 67 (GAD67), a key enzyme for GABA synthesis in animals, is influenced by gut microbiota which regulates gene expression in the gastrointestinal tract (22). In peripheral tissues, pancreatic islets have the highest GABA content (11), primarily derived from pancreatic β-cells (23). Gut microbiota could directly affects pancreatic β-cells express GAD67 to produce GABA (24). WB (**Figures 4C, G**) and IF results (**Figure 4K**) indicated a significant decrease in GAD67 expression in pancreatic β-cells of obH mice compared to obNC mice, while obHA mice treated with antibiotics exhibited a significant increase. The ileum and colon, primary habitats for gut microbiota, are capable of GABA production (25, 26). Our analysis of GAD67 expression in the ileum (**Figures 4D, H**) and colon (**Figures 4E, I**) of obH mice revealed no significant differences compared to other groups. These results suggest that the reduction in GABA levels induced by the HH environment is likely not attributable to changes in the ileum and colon. Research indicates that gut microbiota can directly modulate hypothalamic GABA, influencing metabolism (27). However, our results showed no statistical change in hypothalamic GAD67 expression among the groups (**Figures 4F, J**). Therefore, the reduction in GABA levels caused by the HH environment is likely not driven by changes in the hypothalamus. These findings suggest that the elevation of blood glucose in a HH environment is primarily caused by the reduction of GABA levels, which in turn is mainly due to the decreased expression of GAD67 in pancreatic β-cells.

GABA synthesized by pancreatic β-cells suppresses GC secretion *via* upregulation of GABA_A_β3 receptors on pancreatic α-cells (12, 21). Our investigation utilized WB (**Figure 4L**) and IF (**Figure 4M**) to assess the expression of GABA_A_β3 receptors on pancreatic α-cells. The results indicated a significant decrease in GABA_A_β3 receptor expression in the pancreatic α-cells of obH mice compared to obNC mice (**Figures 4L, M**, *P <*0.01). Notably, this downregulation was counteracted in obHA mice, where GABA_A_β3 receptor levels were significantly elevated (**Figures 4L, M**, *P <*0.05). The findings indicate that diminished GAD67 expression in pancreatic β-cells within HH environment leads to decreased GABA levels. This reduction in GABA subsequently results in lower expression of GABA_A_β3 receptors on pancreatic α-cells, ultimately contributing to an increase in GC and FBG.

## High humidity induces increased expression of the pancreatic MDP-NOD2 pathway, which inhibits GABA synthesis and promotes GC secretion

**Figure 5:**
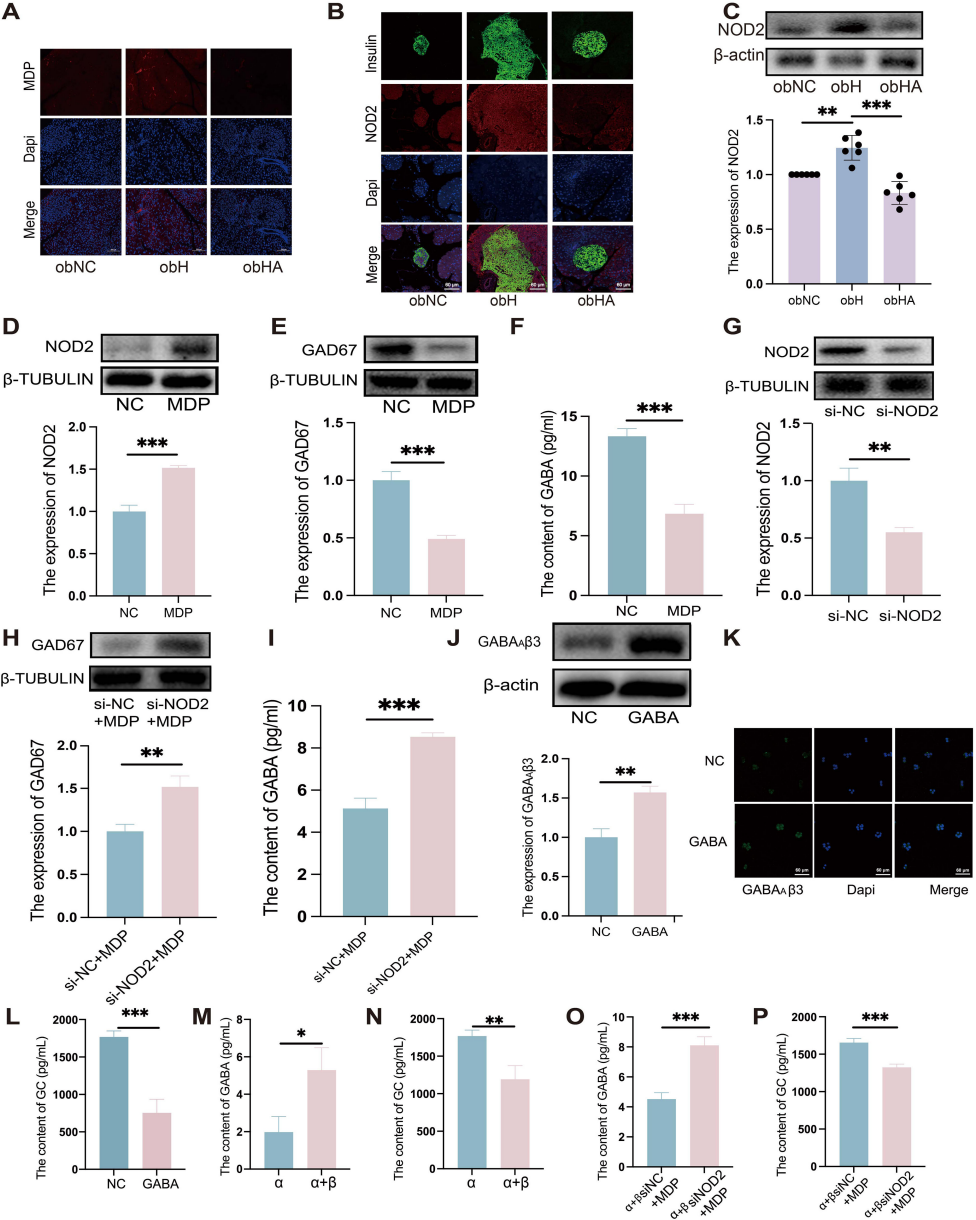
High humidity induces increased expression of the pancreatic MDP-NOD2 pathway, which inhibits GABA synthesis and promotes GC secretion. **(A)** Visualization of MDP in pancreatic tissue, with scale bars indicating 100μm. **(B)** IF staining of NOD2 in pancreatic β-cells. Pancreatic β-cells are marked in green, NOD2 in red, and nuclei with Dapi in blue. Scale bars = 60μm. **(C)** Western blot analysis of NOD2 expression in various groups (n = 6). obNC VS obH was analyzed by Welch’s t test, t=5.303, *P* =0.003. obH VS obHA was analyzed by Welch’s t test, t=6.568, ***P < 0.001, **P < 0.01. **(D, E)** Western blot analysis of NOD2 **(D)** and GAD67 **(E)** in MIN6 cells post-MDP treatment. n = 3, D was analyzed by student’s two-tailed t test, t=11.38, *P* =0.0003. **(E)** was analyzed by student’s two-tailed t test, t=10.79, *P* =0.0004. ***P < 0.001. **(F)**: GABA levels in MIN6 cells treated with MDP. n = 3, F was analyzed by student’s two-tailed t test, t=11.11, *P* =0.0004. ***P < 0.001. **(G)** Western blot analysis of NOD2 in MIN6 cells post-NOD2 knockdown. n = 3, G was analyzed by student’s two-tailed t test, t=6.635, *P* =0.0027. **P < 0.01. **(H)**: Western blot analysis of GAD67 in MIN6 cells post-NOD2 knockdown and MDP treatment. n = 3, **(H)** was analyzed by student’s two-tailed t test, t=5.904, *P* =0.0041. **P < 0.01. **(I)** Quantification of GABA levels in MIN6 cells and NOD2-knockdown MIN6 cells treated with MDP. n = 3, **(I)** was analyzed by student’s two-tailed t test, t=11.04, *P* =0.0004. ***P < 0.001. **(J)** Western blots of GABA_A_β3 in α-TC1–6 cells treated with GABA. n = 3, **(J)** was analyzed by student’s two-tailed t test, t=7.29, *P* =0.0019. **P < 0.01. **(K)** IF staining of GABA_A_β3 in α-TC1–6 cells GABA treatment, with green indicating GABA_A_β3 and blue for Dapi. Scale bars=60μm. **(L)** Measurement of GC levels in α-TC1–6 cells post-GABA treatment. n = 3, L was analyzed by student’s two-tailed t test, t=8.854, *P* =0.0009. ***P < 0.001. **(M-P)** GABA **(M)** and GC **(N)** levels in co-cultured MIN6 and α-TC1–6 cells. GABA **(O)** and GC levels **(P)** in co-cultured α-TC1–6 and NOD2 knockdown MIN6 cells treated with MDP. n = 3. M was analyzed by student’s two-tailed t test, t=3.938, *P* =0.017. **(N)** was analyzed by student’s two-tailed t test, t=5.011, *P* =0.0074. **(O)** was analyzed by student’s two-tailed t test, t=8.731, *P* =0.0009. **(P)** was analyzed by student’s two-tailed t test, t=8.211, *P* =0.0012. ***P < 0.001, **P < 0.01, *P < 0.05. Data are shown as means ± SD. GABA, Gamma-Aminobutyric Acid; GAD67, Glutamate Decarboxylase 67; GABA_A_β3, Gamma-Aminobutyric Acid Receptor Subunit Beta-3; NOD2, Nucleotide-binding Oligomerization Domain-containing Protein 2.

Above results indicate that the HH environment could cause damage to the intestinal barrier. Gut microbes may then translocate through the compromised intestinal barrier into other tissues and organs, such as the pancreas (28). Our findings revealed the high MDP in the pancreatic islets of obH mice, whereas obHA mice exhibited significantly reduced MDP levels (**Figure 5A**). We also observed increased NOD2 expression in the pancreatic β cells of obH mice compared to obNC mice, which was reduced in obHA mice (**Figures 5B, C**, *P* < 0.01). This suggests that HH environment, by altering gut microbiota entry into the pancreatic islets, activates the MDP-NOD2 pathway in obH mice. In line with this results, MIN6 cells treated with MDP showed enhanced NOD2 expression (**Figures 5D, P** < 0.001). However, GAD67 expression (**Figure 5E**, P < 0.001) and GABA content were lower in MDP-treated MIN6 cells compared to control cells (**Figure 5F**, P < 0.001). To further explore NOD2’s role in MDP-induced reduction of GABA, we inhibited NOD2 expression in MIN6 cells using siRNA (si-NOD2) (**Figure 5G**, P < 0.01) and subsequently stimulated these cells with MDP. The inhibition of NOD2 protected against the MDP-induced reduction in GABA synthesis, indicating that MDP suppresses GABA synthesis in MIN6 cells through NOD2 (**Figures 5H, I**, *P* < 0.01). These results underscore the critical role of the MDP-NOD2 pathway in HH environment-induced alterations in GABA synthesis.

In line with *in vivo* results, our *in vitro* experiments confirmed that GABA_A_β3 expression was upregulated (**Figures 5J, K**, *P* < 0.01), while GC secretion was downregulated when treated with GABA (**Figure 5L**, P < 0.001).To further validate whether the reduction in GABA synthesis in MIN6 cells, induced by MDP, could lead to increased GC secretion in α-TC1–6 cells, co-culture experiments were conducted. Compared to α-TC1–6 cells alone, co-cultured α-TC1–6 and MIN6 cells showed a significant increase in GABA levels (**Figure 5M**, P *<*0.05) and a decrease in GC levels (**Figure 5N**, P *<*0.01), indicating that GABA synthesized by MIN6 cells can suppress GC secretion in α-TC1–6 cells. Furthermore, when α-TC1–6 cells were co-cultured with siRNA-mediated NOD2-knockdown MIN6 cells and stimulated with MDP, an increase in GABA levels (**Figure 5O**, P *<*0.001) and a decrease in GC levels (**Figure 5P**, P *<*0.001) were observed compared to α+βsiNC+MDP cells. These *in vitro* findings suggest that MDP inhibits GABA synthesis in MIN6 cells through NOD2, consequently leading to an increase in GC secretion by α-TC1–6 cells.

## Discussion

Numerous studies have documented the detrimental health effects associated with HH, with a particularly pronounced impact on diabetes (2, 29). Despite these findings, the specific mechanisms by which HH influences T2DM remain poorly understood, underscoring the critical need for research in this area. Previous research by our team suggests that the hot and humid environment may influence the expression of GLP-1 through alterations in the gut microbiota, particularly involving the genus *Alistipes*. Although in this study we found an increase in the abundance of the genus *Alistipes* in humid environments compared to the normal group, T2DM patients in HH environment exhibited elevated GC and FBG levels without significant changes in GLP-1 and other hormones. Therefore, we hypothesize that the changes in GC in HH environments are a significant factor contributing to the elevation of FBG. Moreover, Alterations in gut microbial composition can increase gastrointestinal tract permeability, leading to dysregulation of glucose and lipid metabolism, which are key processes in metabolic diseases such as T2DM (30). Dysfunctional gut barriers, marked by the loss of tight junction proteins and increased permeability, are commonly observed in T2DM. This “leaky gut” allows microbes and their metabolites to translocate, disrupting blood sugar regulation (14).

As a commensal gut bacterium in mammals, *g_Akkermansia* and *Parabacteroides* are linked to the metabolism of blood lipids and glucose (31, 32). The presence of these bacteria in both *ob/ob* mice and human T2DM patients under HH environment highlights similar microbial shifts due to environmental factors. Furthermore, increases in serum levels of Zonulin and IFABP, biomarkers of tight junction integrity, were noted in T2DM patients in HH environment, indicating compromised gut barrier function. Moreover, we confirmed that germ-free mice gavaged with fecal samples from HH mice displayed significantly increased FBG and GC, along with impaired gut barrier function. This suggests that the gut microbiota modulated by an HH environment could promote T2DM through a damaged intestinal mucosal barrier in germ-free mice exposed to such conditions. Similarly, *ob/ob* mice in an HH environment exhibited increased FBG, GC, and populations of the genus *Alistipes*, *Parabacteroides*, and *Akkermansia*, along with reduced expression of the tight junction protein ZO-1. Treatment with antibiotics reversed these changes. Collectively, our findings indicate that the HH environment promotes the progression of T2DM by impairing gut barrier function.

Research has demonstrated that GABA can inhibit GC secretion, thereby aiding in the control of blood glucose levels in T2DM (21). Our study found that patients with T2DM, those sampled under condition of HH exhibited significantly lower GABA levels compared to those sampled under less HH environment. Previous studies have indicated that GABA enhances β cell proliferation and reduces blood glucose levels (12). Consistent with these findings, our research revealed that HH markedly decreased GABA content in T2DM mice, while the removal of gut microbiota was found to increase GABA levels. Notably, the highest concentration of GABA in peripheral tissues is observed in the islets (33), likely correlating with the high expression of GAD67 in β cells (34). We discovered that HH inhibited the expression of GAD67 in pancreatic β-cells. However, this effect was alleviated by removing the gut microbiota and repairing the damaged intestinal barrier. Interestingly, this phenomenon was not observed in the ileum, colon, or hypothalamus. These results suggest that HH environment can reduce GABA synthesis in pancreatic β-cells, and this effect is mediated through the restoration of the damaged intestinal barrier.

In our study, we observed a significant reduction in GABA_A_β3 receptor expression in the α-cells of the obH group, accompanied by an increase in GC levels. However, after restoring the damaged intestinal barrier, there was a notable increase in GABA_A_β3 receptor expression and a concurrent decrease in GC levels. These findings indicate that the intestinal barrier plays a critical role in the HH-induced elevation of GC levels. Our results also show that in the obH group, intestinal mucosal permeability is increased, and the MDP-NOD2 signaling pathway in pancreatic beta cells is activated. These findings suggest that HH may induce increased intestinal mucosal permeability, allowing gut microbiota to enter pancreatic beta cells and subsequently activate the MDP-NOD2 signaling pathway. Consistent with our *in vivo* findings, GABA was found to promote GABA_A_β3 receptor expression in α-TC1–6 cells, thereby inhibiting GC secretion. We also found that the GABA content in the cell culture medium increased and GC content decreased after co-culturing, indicating that GABA synthesized by β-cells can inhibit GC secretion by α-cells. Furthermore, we co-cultured α-TC1–6 cells with knocked-down NOD2 expression and MIN6 cells, and then stimulated them with MDP. Compared to the α+βsiNC+MDP group, the α+βsiNOD2+MDP group exhibited increased GABA levels and decreased GC levels. Co-culture experiments revealed that MDP could inhibit GABA synthesis in pancreatic β-cells *via* the NOD2 pathway, leading to increased GC secretion from α-cells. Moreover, our study has several limitations. First, we did not control for potential confounding variables such as seasonal variation in dietary intake among patients with T2DM. Second, the sample size of our study may be insufficient, and we plan to expand the sample size in future research. Finally, although we used ABX to deplete gut microbiota in this study, future investigations using germ-free mice are warranted to validate and extend our findings.

In summary, these studies reveal the impact of a HH environment on T2DM and the underlying mechanisms, which will aid in the development of intervention strategies.

## Materials and methods

### Ethical approval

The study was conducted in accordance with applicable regulations, and informed consent was obtained from participants. The trial has been registered with the Chinese Clinical Trial Registry under the registration number “ChiCTR1900023995”.

Experimental procedures were conducted following approval by the Animal Experimental Ethical Committee of the Traditional Chinese Medicine Hospital of Zhongshan (Approval No. 2021064) and in compliance with the National Institutes of Health Guide for the Care and Use of Laboratory Animals.

### Study participants

We enrolled a total of 20 patients diagnosed with T2DM. The project was approved by the Medical Ethics Committee of Hainan Traditional Chinese Medicine Hospital. Participants provided signed informed consent before joining the study. The participants’ sex was confirmed through self-reporting, appearance, and identity card information. We collected the daily mean, maximum, and minimum temperatures, as well as the daily relative humidity from August 30, 2020, to February 1, 2021, from the Haikou weather stations of the China Meteorological Data Service Centre (http://data.cma.cn/). These measurement values were grouped according to seasonal humidity levels. The data served as a reference to determine the start and end dates of each environmental exposure phase. Exposure conditions were classified as follows: Rainy Season (August 30, 2020 – October 30, 2020; humidity: 80-90%; T2DMH, n=10) and Dry Season (December 1, 2020 – February 1, 2021; humidity: 50-70%; T2DMnH, n=10). Patients recruited during the rainy season were assigned to the T2DMH group, while those recruited during the dry season were assigned to the T2DMnH group. Serum and fecal samples were collected on the first day of enrollment. Patients were enrolled consecutively. The inclusion criteria for participants were as adhered to the criteria established by the World Health Organization (WHO), encompassing: (1) a random blood glucose (venous plasma glucose) level equal to or exceeding 11.1 mmol/L; (2) a fasting plasma glucose level (after at least 8 hours of fasting) equal to or surpassing 7.0 mmol/L; and (3) a 2-hour venous plasma glucose level of 11.1 mmol/L or higher during an oral glucose tolerance test.

### Mice treatment

Six male BALB/c mice (6 weeks old) were randomly divided into two groups and housed in sterile, autoclaved cages (n=3), with ad libitum access to irradiated chow and acidified water. The housing environment was controlled at a temperature of 25 ± 2°C and average humidity levels between 50-70%, complemented by a 12-hour light/dark cycle. Both water and rodent chow were made available ad libitum. For the study, the mice were divided into two groups: a high humidity group (HH) and a normal control group (NC), with the latter receiving no specific treatment. The HH group was subjected to an environment of 80-90% relative humidity at 25 ± 2°C for 14 days, facilitated by an artificial climate box (model RXZ-158A, Ningbo Jiangnan Instrument Factory, China). The raw data of 16S rDNA sequencing was accessed in BioProject database (PRJNA1032397).

In the FMT experimental protocol (**Figure 1A**), mice were randomly allocated into two distinct groups: the treatment group (DF, n=3), receiving FMT from mice subjected to four weeks of high-humidity exposure, and the control group (NF, n=3), receiving FMT from mice kept in standard environmental conditions. Both groups were accommodated in sterile cages within a controlled setting at a temperature of 25 ± 1°C and a relative humidity ranging from 60 to 65%. Fresh fecal samples (200 mg) collected from mice either from normal or high-humidity conditions after four weeks were dissolved in 5 mL of saline solution. This mixture was vigorously agitated for 3 minutes and then left to settle for 2 minutes under gravity. The FMT procedure involved gavage administration of 200 μL of the resultant supernatant to the DF and NF groups, carried out over a two-week period with a total of six administrations. The raw data of 16S rDNA sequencing was accessed in BioProject database (PRJNA1032695).

For antibiotic treatment, an antibiotic cocktail (ABX) was prepared by dissolving 0.5 g vancomycin, 0.5 g metronidazole, 1 g penicillin, and 1 g neomycin in 1 liter of filtered distilled water (35). In this study, eighteen male *ob/ob* mice (B6.V-Lepob*ob/ob*/JRj), which exhibit diabetic phenotypes, were randomly assigned to three groups (n = 6). These mice were further categorized into six groups: C57BL/6 control (C57NC), *ob/ob* control (obNC), high-humidity *ob/ob* (obH), high-humidity *ob/ob* with ABX treatment (obHA). The obH and obHA groups were exposed to the aforementioned HH environment. High-humidity: relative humidity:80-90%, temperature:25 ± 2°C, 14 days. The raw data of 16S rDNA sequencing was accessed in BioProject database (PRJNA1047322).

### MR

Mendelian Randomization (MR) analyses were conducted using the TwoSampleMR package (v.0.5.7) in R. This study primarily focused on elucidating the causal relationships between gut microbiota and T2DM. The exposure GWAS data for the gut microbiome was obtained from MiBioGen consortium (https://mibiogen.gcc.rug.nl/). T2DM dataset finn-b-E4_DM2KETO, ieu-a-23 dataset were acquired from MRC-IEU GWAS Database (https://gwas.mrcieu.ac.uk). Initially, linear regression was conducted for each genetic variant using a less stringent significance threshold (*P* < 1 × 10^-5^) to screen potential instruments, as suggested by previous studies (36, 37). This approach aimed to reduce potential bias in the MR analysis. Subsequently, SNPs in linkage disequilibrium (LD) were excluded. LD clumping was performed using a window size of 5000 kb and an r² threshold of 0.01. To evaluate the strength of the instruments and minimize weak instrument bias, *F*-statistics were calculated, with values greater than 10 indicating sufficient instrument strength.

To ensure the robustness of our MR-derived causal inferences, multiple sensitivity analyses were conducted. These included the assessment of horizontal pleiotropy *via* the MR-Egger intercept (*P* > 0.05), evaluation of heterogeneity using Cochran’s Q test (*P* > 0.05) (38). In MR analyses, key statistical metrics such as odds ratios (ORs) are crucial for evaluating associations between genetic variants and specific traits or diseases. An OR greater than 1 suggests a positive association; less than 1 indicates a negative association, while an OR equal to 1 denotes no association. The magnitude of deviation from 1 is indicative of the strength of the correlation. Beta values are employed to describe the magnitude and direction of associations in continuous traits. The standard error (SE) is used to quantify the uncertainty in these estimations. The 95% confidence interval of the odds ratio provides a range of plausible values for the OR, where the inclusion of the value 1 indicates a non-significant association, and exclusion signifies statistical significance. The initial phase of two-sample MR aimed to identify and prioritize gut microbiota significantly associated with T2DM.

### Cell culture

MIN6 cells, were procured from Servicebio (China). The cells were maintained in a culture medium comprising RPMI-1640 medium (Gibco, USA), enriched with 10% fetal bovine serum (FBS) (Gibco, USA), and supplemented with 5 μmol/L 2-mercaptoethanol (Maklin, China). For the MDP stimulation experiments, cells at 90% confluence were treated with MDP sourced from Sigma (USA), at a final concentration of 500 μg/mL, and incubated for 12 hours. The targeted knockdown of NOD2 in MIN6 cells was achieved using a specific single-gene siRNA kit provided by Obio Biotechnology Co., Ltd. (Obio, China).

α-TC1–6 cells, also obtained from Servicebio (China), were cultured in high-glucose Dulbecco’s Modified Eagle Medium (DMEM) (Gibco, USA), fortified with 10% FBS (Gibco, USA). Upon reaching 90% confluence, these cells were treated with GABA procured from Sangon Biotech (China). The administered concentration of GABA was set at 20nM, and the cells were incubated for a period of 24 hours. For co-culture experiments, α-TC1–6 and MIN6 cells were seeded at a 2:1 ratio in 6-well plates. After a 24-hour period to allow for cell adhesion, the supernatant was collected for subsequent measurements of GC and GABA levels, which were quantified using ELISA.

### 16S rDNA

DNA extraction from stool samples was carried out using the TIANamp Stool DNA Kit (TIANGEN), adhering to the protocols established in previous studies (39). The bead-beating method was employed for DNA collection, followed by extraction using phenol and chloroform, and subsequent dissolution in Tris-EDTA (TE) buffer. The quality and concentration of the extracted DNA were verified via electrophoresis on 1% agarose gels. Based on these concentration measurements, DNA samples were diluted to a final concentration of 1 ng/μl using sterile water.

Amplification of the variable regions 3 and 4 (V3–V4) of the bacterial 16S rDNA gene utilized specific primers 341F and 806R (341: CCTAYGGGRBGCASCAG; 806: GGACTACNNGGGTATCTAAT), incorporating barcoding techniques (40, 41). PCR reactions were conducted in 30μL volumes, comprising 15μL of Phusion^®^ High-Fidelity PCR Master Mix (New England Biolabs), 0.2μM of each primer, and approximately 10 ng of template DNA. The PCR thermal profile included an initial denaturation at 98°C for 1 minute, 30 cycles of denaturation at 98°C for 10 seconds, annealing at 50°C for 30 seconds, and elongation at 72°C for 60 seconds, culminating with a final extension at 72°C for 5 minutes. Post-PCR, an equal volume of 1X loading buffer (with SYBR green) was mixed with the PCR products and analyzed via electrophoresis on 2% agarose gels, selecting samples with a prominent main band between 400–450 bp for subsequent procedures. PCR products were pooled at equidensity ratios and purified using the Qiagen Gel Extraction Kit (Qiagen). Library construction for sequencing followed the TruSeq^®^ DNA PCR-Free Sample Preparation Kit (Illumina) protocol, with the addition of index codes. Library quality was ascertained using the Qubit@ 2.0 Fluorometer (Thermo Scientific) and Agilent Bioanalyzer 2100 system. Sequencing was performed on an Illumina NovaSeq6000 platform, genusting 250bp paired-end reads.

For α-diversity analysis, the vegan package (version 2.6.4) in R was utilized to calculate diversity indices. Principal Coordinates Analysis (PCoA) and Non-Metric Multidimensional Scaling (NMDS) were employed for β-diversity analysis. Functional categorization of microbial communities based on KEGG pathways was conducted using PICRUSt. The LefSe (LDA Effect Size) analysis identified significantly different taxa or species between groups, using linear discriminant analysis (LDA) based on taxonomic composition, with species showing an LDA score greater than 2 being considered as statistically significant biomarkers. Alpha and beta diversity analyses were conducted using the R software package (version 4.2.3).

### MRM targeted metabolomics

Upon retrieval from -80°C storage, samples were gradually thawed at 4°C. Each 200 μL sample aliquot was combined with 800 μL of prechilled methanol-acetonitrile solution (1:1, v/v). The mixture underwent vigorous vortexing for 60 seconds, followed by a 1-hour protein precipitation phase at -20°C. Centrifugation was conducted at 14,000 × g for 20 minutes at 4°C, after which the supernatant was isolated and subsequently freeze-dried. Samples were then stored at -80°C until further analysis.

Sample separation was executed using an Agilent 1290 Infinity LC ultra-high-performance liquid chromatography system (Agilent, USA). The mobile phase comprised solvent A (25 mM ammonium formate with 0.1% formic acid) and solvent B (acetonitrile with 0.1% formic acid). Samples were preserved at 4°C in the autosampler, and the chromatography column was maintained at a steady temperature of 45°C. The flow rate was set to 300 μL/min, and the injection volume was 2 μL. The gradient programming was as follows: a) 0–18 min, solvent B varied linearly from 90% to 40%; b) 18-18.1 min, a swift transition of solvent B from 40% back to 90%; c) 18.1–23 min, solvent B sustained at 90%. Quality control (QC) samples were integrated systematically into the sample queue to ensure the system’s stability and reproducibility. A mix of neurotransmitter standards was also included for precise retention time alignment.

A 5500 QTRAP mass spectrometer (AB SCIEX, Canada) was employed for mass spectrometric analysis in positive ion mode. The ESI source settings were adjusted as follows: a) Source temperature at 450°C; b) Ion Source Gas1 (Gas1) at 60; c) Ion Source Gas2 (Gas2) at 60; d) Curtain gas (CUR) at 30; e) Ion Spray Voltage Floating (ISVF) set to 5000 V. Peak areas and retention times were extracted using Multiquant software, with neurotransmitter standards facilitating retention time correction and metabolite identification.

### ELISA

ELISA kits for mouse GABA (CEA900Ge; Elabscience), glucagon (E-EL-M0555, Elabscience), and insulin (E-EL-M1382; Elabscience) were used. Glucagon and insulin were performed using serum samples. And the GABA was performed using plasma samples. The ELISA was performed according to the manufacturer’s instructions. The standard curves were plotted for each experiment.

### Immunofluorescence

Pancreatic tissues were harvested from fasted mice (approximately 16 hours of fasting), then subjected to formalin fixation and paraffin embedding for histological analysis. Tissue sections, 6μm thick, were mounted on positively charged slides and dewaxed. Antigen retrieval involved immersing the slides in 0.01 M sodium citrate buffer (pH 6.0) for 25 minutes using a steam-based heating method. For immunofluorescence staining, the sections were first blocked using normal goat serum for 30 minutes, then incubated sequentially with primary antibodies at 4°C overnight, followed by fluorophore-conjugated secondary antibodies for 2 hours at room temperature. After staining, slides were counterstained with DAPI and mounted using Fluoromount-G. Confocal microscopy images were captured using a Zeiss imaging system.

For cellular immunofluorescence, cells cultured on cover slips were initially rinsed with cold PBS and then fixed with 4% paraformaldehyde for 15 minutes at 4°C. This was followed by three washes with cold PBST (PBS with 0.1% Triton X-100). Blocking was done using 10% goat serum in PBST, after which cells were incubated with primary antibodies overnight at 4°C. This step was succeeded by incubation with fluorophore-conjugated secondary antibodies for 2 hours at room temperature. The cover slips were washed three times for 5 minutes with PBST between each incubation step. Normal goat serum was used as a blocking reagent throughout the antibody incubation processes. Finally, cells were counterstained with DAPI and imaged using a Zeiss imaging system.

### MDP immunofluorescence staining

Post-wash, tissues were promptly fixed in a DEPC-treated fixative solution for a minimum of 12 hours. Subsequent to fixation, a graduated ethanol series was employed for tissue dehydration, followed by paraffin embedding and microtome sectioning. The obtained sections were floated in a water bath and then transferred onto slides, which were subsequently baked at 62°C for 2 hours.

For deparaffinization and rehydration, sections underwent treatment with xylene I and II for 15 minutes each, followed by two consecutive 5-minute immersions in 100% ethanol. Post air-drying, the slides were submerged in DEPC water. Tissue sections, based on fixation duration, were then boiled in a retrieval solution for 5 minutes and cooled naturally. A hydrophobic barrier was created around the sections using a hydrophobic pen, followed by a 15-minute incubation at 37°C with 20 μg/ml proteinase K. The sections were then washed thrice with PBS for 5 minutes each.

For hybridization, sections were first incubated with a prehybridization solution at 37°C for an hour. The prehybridization solution was then replaced with a hybridization solution containing the EUB338 probe at a 1 μM concentration, and the sections were hybridized overnight at 40°C in a specialized chamber. Post-hybridization, sections were washed with 2× SSC at 37°C for 10 minutes, followed by two 5-minute washes in 1× SSC at 37°C, and a 10-minute wash in 0.5× SSC at room temperature. In cases of non-specific hybridization, a formamide wash was performed. Subsequently, sections were stained with DAPI solution, incubated in the dark for 8 minutes, rinsed, and mounted using an anti-fade mounting medium. Image acquisition was conducted using Nikon imaging systems, capturing high-resolution visual data for analysis.

### Quantitative reverse-transcription PCR

RNA was isolated from pancreatic islets using TRIzol reagent (Thermo Fisher Scientific, USA), adhering strictly to the manufacturer’s protocol. The reverse transcription of mRNA was carried out using the PrimeScript™ RT Reagent Kit with gDNA Eraser (TAKARA, Japan). Real-time PCR assays were conducted in triplicates using TB Green^®^ Premix Ex Taq™ II (Tli RNaseH Plus) on a CFX96 Real-Time PCR Detection System. The PCR amplification protocol commenced with an initial denaturation at 95°C for 30 seconds, followed by 40 amplification cycles, each consisting of a 5-second denaturation at 95°C and a 30-second annealing/extension phase at 60°C. Melting curve analysis was employed post-amplification to verify the specificity of each PCR product. Gapdh, a housekeeping gene, was utilized as an internal control for normalization purposes. The relative mRNA expression levels were quantified using the Delta-Delta Ct method, which involves a comparative analysis between the target gene expression and the Gapdh control. Primer sequences used were as follows: for NOD2, forward: CAATCGCTGGACGCCCTTCTG, reverse: TGGCAAACTCTTCTCCTTGGATGTC; for GAD67, forward: CATCATGGCGGCTCGGTACAAG, reverse: GTGACTGTGTTCTGAGGTGAAGAGG.

### Western blot

Total protein was isolated from either tissue homogenates or cellular lysates, using RIPA buffer (Beyotime Biotechnology, China), supplemented with 1× concentrations of protease and phosphatase inhibitors. Protein concentrations in these tissue extracts were quantified via the BCA Protein Assay Kit (Thermo Fisher Scientific, USA). Subsequently, equivalent protein amounts from each sample group were resolved by SDS-PAGE and then electro-transferred onto PVDF membranes. These membranes were initially blocked with a 5% BSA solution in 1× TBST for an hour at ambient temperature, followed by an overnight incubation at 4°C with primary antibodies targeting NOD2 (1:1000 dilution) (SAB, USA), GAD67 (1:1000 dilution) (Abcam, USA), and GABA_A_β3 (1:1000 dilution) (Abcam, USA). Post antibody incubation, membranes were washed and further incubated with appropriate HRP-conjugated secondary antibodies (either anti-rabbit or anti-mouse, 1:5000 dilution, Abcam, USA) for an hour at room temperature. Protein band visualization was achieved using the SuperSignal West Femto Maximum Sensitivity Substrate (Thermo Fisher Scientific, USA). As loading controls, β-tubulin (1:2000 dilution) (Affinity, China) and β-actin (1:2000 dilution) (Abcam, USA) were employed. Band intensities were quantitatively analyzed using ImageJ software.

### Statistical analysis

Apart from the gut microbiome data, other results were presented as mean ± standard error of the mean (SD). Statistical significance between mean values was determined using student’s t-test or Welch’s t test for comparisons involving two groups, and one-way ANOVA for data involving more than two groups. Each measurement was derived from distinct samples. Differences were considered statistically significant at a threshold of *p* < 0.05.

## Data Availability

Primary experimental data will be shared upon personal request by the corresponding authors. 16S rRNA gene sequencing data were deposited in the BioProject database (Accession numbers are PRJNA1047322, PRJNA1032397 and PRJNA1032695, and are publicly available.
