# Supplementary material for: High humidity environment increases FBG by impairing the intestinal barrier
Source: Front Immunol. 2025 Aug 27;16:1625609. doi: 10.3389/fimmu.2025.1625609 (PMC12420254; doi:10.3389/fimmu.2025.1625609)
Supplement: Supplementary file 3 [file Table2.docx]

Supplementary Material

# Supplementary Data


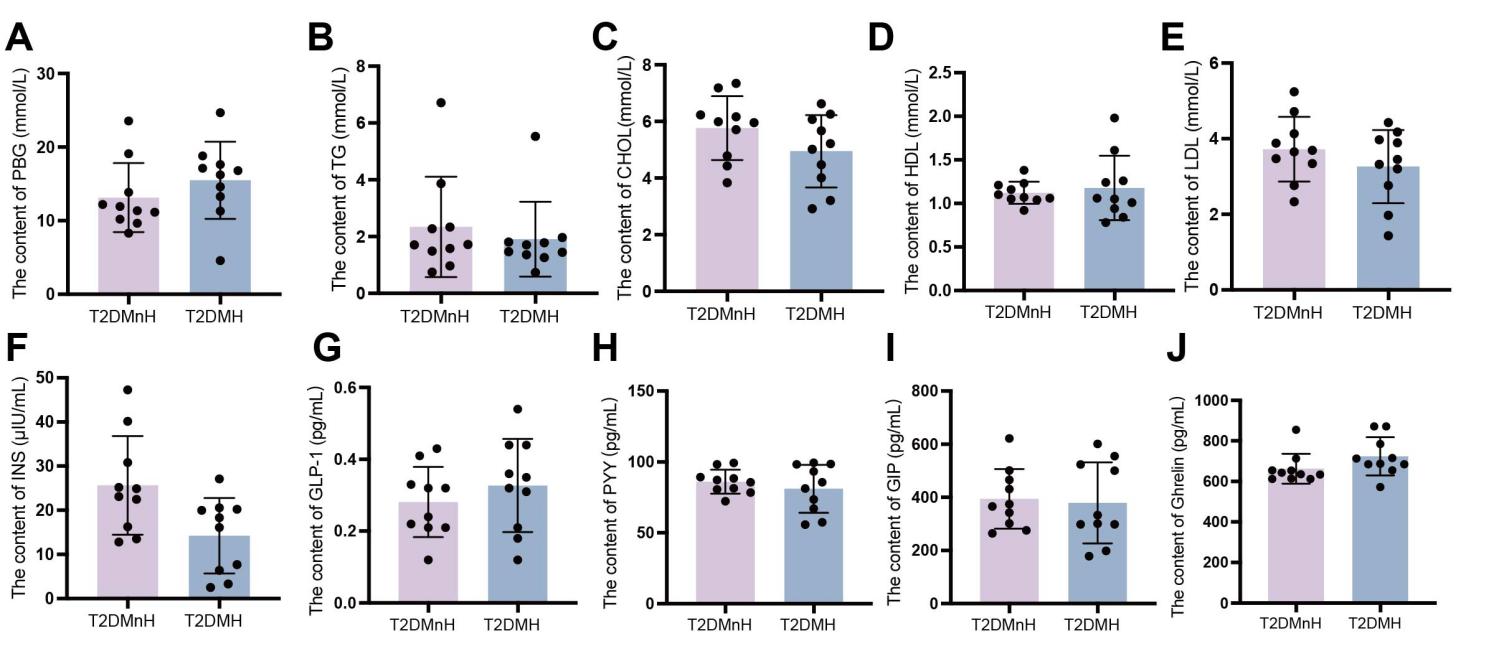


**Fig 1:** Blood was collected for PBG (A), TG (B), CHOL (C), HDL (D) LDL (E), INS (F), GLP-1 (G), PYY (H), GIP (I) and Ghrelin (J). Data are shown as means ±SD.


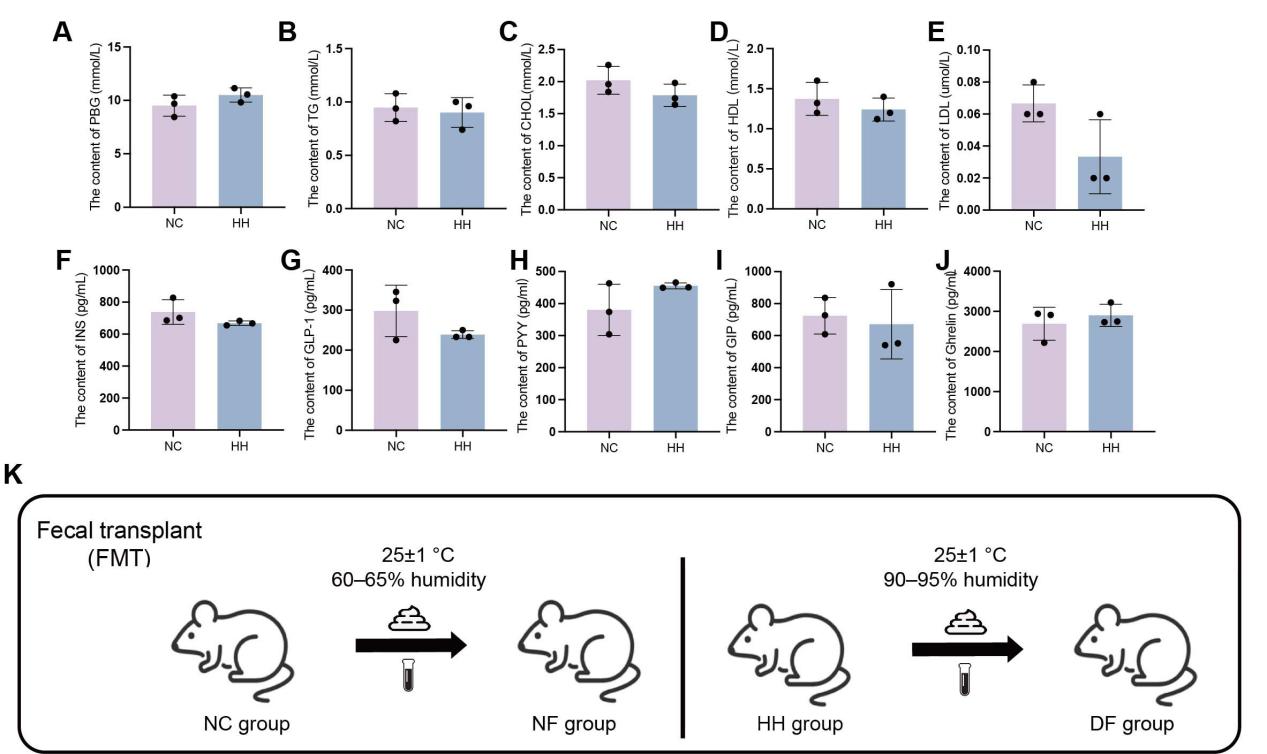


Supplementary Fig 2: Blood was collected for PBG (A), TG (B), CHOL (C), HDL (D), LDL (E), INS (F), GLP-1 (G), PYY (H), GIP (I) and Ghrelin (J). K: The animal experimental protocol for FMT. Data are shown as means ±SD.


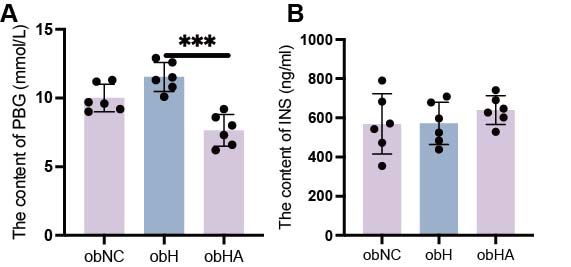


Supplementary Fig 3: The content of PBG (A) and INS (B) in different groups.


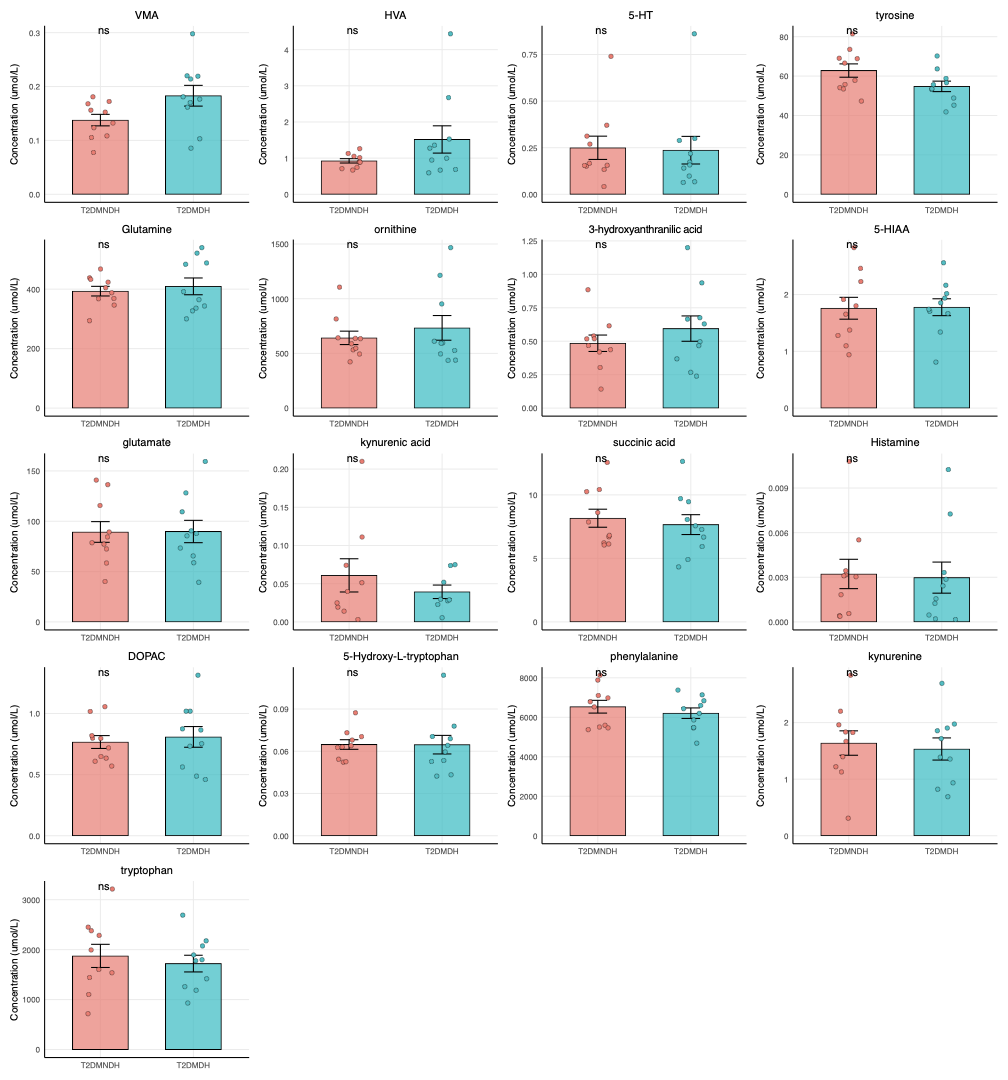


Supplementary Fig 4: Results from MRM analysis of neurotransmitter levels in the blood of clinical patients ( n = 10). Data are shown as means ±SD.
